# Mineral Stabilization Slows Losses of Peatland Carbon Following Long‐Term Drainage for Agriculture

**DOI:** 10.1111/gcb.70985

**Published:** 2026-07-09

**Authors:** Katy J. Faulkner, Katerina Georgiou, David A. Coomes, Philippa Ascough, Anna Basford, Rodney G. O. Burton, Romy Copley, Emma Keerberg, Alanie Lapina, Kimber Moreland, Christopher Evans, Ross Morrison, Adam F. A. Pellegrini

**Affiliations:** ^1^ Department of Plant Sciences and Conservation Research Institute University of Cambridge Cambridge UK; ^2^ Department of Biological and Ecological Engineering Oregon State University Corvallis Oregon USA; ^3^ NEIF Radiocarbon Laboratory SUERC East Kilbride UK; ^4^ Environmental Consultant Cambridge UK; ^5^ Biological Sciences University of Edinburgh Edinburgh UK; ^6^ Department of Earth System Science, Doerr School of Sustainability Stanford University Stanford California USA; ^7^ UK Centre for Ecology & Hydrology Bangor UK; ^8^ UK Centre for Ecology & Hydrology Wallingford UK

**Keywords:** agricultural land‐use emissions, mineral‐associated organic matter, organic soils, radiocarbon

## Abstract

Cultivated peatlands are a major CO_2_ emission source, but the processes that regulate the decomposition of drained peat are debated, especially as drained peat becomes increasingly shallow. Many cultivated peatlands are underlain by a mineral soil layer. Surface subsidence, oxidation and tillage reduce the peat thickness, which intermixes peat with minerals as peat is lost. A key question is whether this mixing can reduce emissions by making soil organic carbon (SOC) more stable by transforming particulate organic carbon (POC)—which dominates the carbon stock in deep drained peatlands and is readily accessible to microbial decomposers—into mineral‐associated organic carbon (MAOC), which is less accessible to decomposers. To explore this question, we surveyed ten sites across the East Anglian Fens in England, a once extensive (~3900 km^2^) peatland landscape that has been drained for cultivation since the mid‐seventeenth century. We used variation in cultivation to establish a peat loss gradient to evaluate how topsoil SOC (0–40 cm) and its forms change as peat is lost. As the peat was lost, the soils became rich in minerals (rising from 46% to 88% silt+clay content), resulting in an initial 11‐fold rise in newly‐formed MAOC, but a monotonic decline and near‐total loss of POC. POC turnover times were 3158 ± 62 years, indicative of peat, and POC was always older than MAOC; consequently, microbially‐processed peat along with gradual contributions of recently fixed carbon were sources of MAOC. A four‐month laboratory incubation showed that the MAOC:POC ratio was negatively correlated with respiration. We conclude that long‐term carbon retention via MAOC formation has the potential to reduce carbon loss from degraded, mineral‐mixed peatlands. However, because this MAOC pool is itself vulnerable to loss under continued agricultural drainage, this mechanism is expected to slow rather than halt long‐term soil carbon loss from drained peatlands.

## Graphical abstract

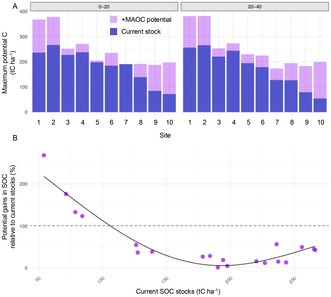

## 1 Introduction

Emissions from agriculture contribute a substantial amount to rising levels of greenhouse gases (GHGs) in the atmosphere (Rosenzweig et al. 2023; Tubiello et al. 2015). The fraction from land‐use change and land management can be on the order of ca. 1/3 of total agricultural emissions (Hong et al. 2021; Tubiello et al. 2015). Cultivation on drained peatlands is one particularly concerning emission source because of the high density of organic carbon in peatlands that has accrued over millennia but can be lost over years to decades following drainage (Hooijer et al. 2012; Page et al. 2011). For example, in 2019, drained organic soils spanned 24 million hectares (Conchedda and Tubiello 2020), and cultivated peat accounted for an estimated 32% share of global cropland greenhouse gas emissions but only 1.1% of total crop kilocalorie production (Carlson et al. 2017). Estimates of carbon losses from cultivated peatlands either rely on the assumption of constant (Department for Environment, Food, and Rural Affairs 2022; Kasimir‐Klemedtsson et al. 1997; Lloyd et al. 2023) or proportionally decreasing (Evans et al. 2016) emissions as peat thickness declines. However, the processes controlling changes in carbon losses as peat depths decline remain unclear.

One important stabilization process may be the vertical intermixing of minerals and peat. Peat‐mineral mixing can occur due to hydrological processes (e.g., flooding, preferential flow pathways, and water‐table fluctuations), biological activity (e.g., roots and soil fauna), and physical disturbances (e.g., subsidence and tillage) (Schothorst 1977; Wang et al. 2021). This intermixing can potentially stabilize residual peat‐derived carbon and reduce some of the total losses during cultivation (Anthony and Silver 2020; Kalisz et al. 2021; Qin et al. 2022; Wang et al. 2016, 2021). Peat‐decomposition products may form more persistent complexes, namely by associating with soil minerals (referred to mineral‐associated organic matter, MAOM) (Anthony and Silver 2020; Georgiou et al. 2025; Torn et al. 1997). In theory, mineral subsoils formed prior to the onset of peat formation have received little carbon inputs because of being waterlogged for millennia and may be under‐saturated with respect to their carbon storage potential. Reconnection of the mineral subsoil with the contemporary carbon cycle following drainage, thinning of the peat layer, and subsequent intermixing with peat via ploughing provide theoretically greater capacity to sorb organic matter (Abramoff et al. 2021; Breure et al. 2025; Cotrufo et al. 2019; Georgiou et al. 2022, 2025; Lavallee et al. 2020). The degree to which this sorption capacity is reached depends on the balance between inputs and losses, and the resulting organic‐carbon loading on minerals. Assuming peat‐derived organic matter is the primary source to the MAOM pool, as peat is decomposed and respired, a portion should theoretically sorb to minerals and become part of the MAOM pool. If soils rich in minerals have little MAOM relative to their theoretical capacity (i.e., mineral deficit), restoring inputs is hypothesized to lead to SOC sequestration into the MAOM pool (Georgiou et al. 2022). Consequently, the relative partitioning of soil organic matter in MAOM versus POM is used as a framework to assess how global change may alter total SOC storage (Prairie et al. 2023; Rocci et al. 2021; Schmidt et al. 2011; Stanley et al. 2024).

The association of organic carbon with minerals is driven largely through microbial decomposition (Lavallee et al. 2020; Sokol et al. 2022), although plant biomass can also directly associate with minerals (Angst et al. 2021; Chang et al. 2024). As microbes decompose the peat and “fresh” (recently photosynthesized) carbon from crops, they die and their necromass and byproducts serve as a “microbial pump” onto the minerals (Zhu et al. 2020). The subsequent association between organic matter and minerals creates a bond that makes it difficult for microbes to subsequently decompose the MAOM (Lavallee et al. 2020). Microbial decomposition is expected to preferentially favor particulate organic matter (POM) (Lavallee et al. 2020), suggesting greater microbial activity when recently drained peats are in early stages of decomposition because POM is high. As POM declines, and the peat layer becomes progressively thinner, decomposition should decline concurrently because the soils become increasingly dominated by MAOM, which makes more of the carbon less accessible to microbial decomposition. Accessibility can be assessed in several ways, but often long‐term soil incubations can provide insight into how different forms of SOC and microbes regulate losses of labile carbon.

Studies of peat–mineral mixing reveal contrasting effects on SOC stability and carbon losses from drained peatlands depending on physico‐chemical interactions with the peat. For example, covering peat with mineral soils can either reduce decomposition of the old peat (Wang et al. 2021) or have no effect (Paul et al. 2024). In another study, siltation across a drained peatland resulted in lower oxidation potential of soil organic matter (SOM) and changes in SOM humification, pointing to lower losses (Kalisz et al. 2021). Mineral soils can promote losses when added to peat; however, when physicochemical changes promote decomposition (Anthony and Silver 2020; Qin et al. 2022). Consequently, it is unclear to what degree soil carbon becomes “stabilized” as peat intermixes with minerals and whether this could constrain either the total amount or the rate of long‐term carbon losses arising from oxidation of the drained peat. Moreover, whether the accrued MAOM constitutes a true “stabilization” of peat in the sense that it is formed from peat versus crops growing on the peat is unclear. However, knowing the source has implications for whether we view stabilization as avoided emissions from peat drainage, a mechanism that attenuates but does not halt peat carbon loss, or a form of new carbon sequestration.

The UK's East Anglian Fenland region (“the Fens”) is an extensive area of lowland peatland used for highly productive agriculture (1500 km^2^), with ca. two‐thirds of the cultivated area (ca. 1000 km^2^) being on peatland that has become degraded (defined as soils with < 40 cm peat and referred to locally as “wasted peat”) (Lloyd et al. 2023; Wells and Wheeler 1999) since large‐scale drainage for cropland agriculture began in the 17th century. The main pathway for peat loss is microbial respiration under aerobic conditions (Evans et al. 2021); these losses can be large, estimated to be 1680 gCO_2_‐eq/m^2^ yr.^−1^ for grasslands and 2090 gCO_2_‐eq/m^2^ yr.^−1^ for croplands, totaling 1.7% of total anthropogenic emissions in the UK (Department for Environment, Food, and Rural Affairs 2022; Rhymes et al. 2023). CO_2_ emissions from these areas generally increase with increasing depth of drained peat, but decline as peat degradation leads to thinner and more carbon‐depleted soils (D'Acunha et al. 2026; Evans et al. 2016, 2021). One mechanism behind this decline is thought to be substrate limitation: as SOC declines, there is less SOC to be decomposed. An alternative mechanism behind the declining emissions could be the introduction of minerals as peat is mixed into the underlying mineral soil horizons. Stabilization of the peat could occur through direct sorption of organic matter to minerals and/or transfer to minerals via the microbial pump (Angst et al. 2021; Chang et al. 2024; Lavallee et al. 2020; Sokol et al. 2022).

These processes produce several predictions related to the biogeochemical changes occurring during peat degradation: (i) as peat degrades and the underlying mineral soil becomes biogeochemically connected via drainage, peat thinning and physical mixing, both the potential for mineral sorption and the amount of carbon associated with minerals should increase; (ii) carbon in degrading peatland soils should switch from being dominated by POC to being increasingly MAOC; (iii) the association between organic carbon and minerals should increase relative to the theoretical carbon‐storage potential of mineral surfaces (i.e., the carbon deficit of minerals) decreases (Abramoff et al. 2021; Georgiou et al. 2025); (iv) as SOC is increasingly associated with minerals, bulk soil decomposability should decline.

We hypothesize that POM in this system is predominately derived from ancient (millennial‐aged) peat. As peat is decomposed by microbes, it is mixed with fresh carbon inputs from crop residues to build microbial biomass. As microbial biomass turns over and forms MAOM, we expect the resulting MAOM to have younger radiocarbon ages than peat‐dominated POM. Thus the “microbial pump” concept can be tested based on the relative radiocarbon ages of the POM and MAOM fractions. As SOC is increasingly associated with minerals, we expect decomposition to decline if the MAOM is truly stabilized and less accessible for microbial decomposition.

## 2 Methods

### 2.1 Study Design

We explored the role of SOC stabilization with minerals across a peat‐degradation gradient in the East Anglian Fenlands, England, UK, with soils spanning an organic matter gradient of 11.1%–54.0% across the 0–40 cm profile depths (i.e., carbon‐rich mineral soils to predominantly humified peat). We focused on 0–40 cm because this captures the layer where “wastage” is most apparent, with the residual peat eventually becoming part of a mineral‐mixed plough layer, and is the depth threshold used in policy to delineate degradation status. However, we characterize soils based on pedogenic surveys that capture the depth/thickness of the peat (Figure S1). Our sampling design was set up in two parts: (i) sites across the fens spanning a range of soil types and mineralogy and (ii) fields within farms containing lower and higher SOC to account for localized management effects.

### 2.2 Site Description

The East Anglian Fens are an area of lowland peat which was widely drained for increasingly intensive agricultural production from the 17th century onwards (Lloyd et al. 2023; Wells and Wheeler 1999). The East Anglian Fens present a unique landscape to evaluate how the mixing between peat and minerals might change the trajectory of soil carbon losses. This is because the degree to which peat has been lost varies both across the entire region as well as within farms. The current peat depth is a function of natural pre‐drainage peat‐depth gradients, presence/absence of roddons under the peat, and time since drainage. Drainage depth is important, but this is unlikely to vary across the Fens given it is almost all drained now to below the base of the peat layer. Thus, our sites do not form a clear stratified gradient of pristine peat overlying mineral soils. Rather, they represent variability in the total SOC content, depth of layers classified as peaty and their mineral content. All the soil profiles are drained. Because it is likely that the areas had varying original peat depths, we do not base our analysis on a strict chronosequence or continuous relationship with peat depth. Rather, we generate a rank‐order of the sites in terms of their total organic matter content combined with detailed soil profile information (Figure S1) to reconstruct a gradient in peat degradation.

Our selected farm sites were in five areas across the fens: Pymoor (52.4549° N, 0.2013° W), Barway (52.3576° N, 0.2670° E), Manea (52.4829° N, 0.1784° E), Earith (52.3539° N, 0.0311° E) and Sawtry (52.4342° N, 0.2820° W). We selected five arable farms for our study which capture the range of agriculturally drained soil types found across the region (i.e., the few remaining fragments of undrained peatland were not included) and which span an organic matter gradient from 11% to 54% and total depth of peat in the profile from 0 to 109 cm. In the cases where peat layers were separated by mineral soils, we would add the thicknesses of only the peat layers. We extensively characterized and classified the soil profiles at each sampling location (Figure S1). The soil types range from histosols to gleysols (World Reference Base for Soil Resources) and from sites with relatively deep peat to “wasted peat” where much of the peat has been lost, to mineral soils (Figure S1). It is difficult to know for certain that the mineral soil sites had peat, so we refer to these as the lowest SOC sites.

We characterized the soil profiles at each sampling site down to 1 m (Figure S1) based on the presence and form of peat, texture, pH, organic content, size fractions, and underlying mineralogy. The sites spanned a large “peaty” gradient. The most intact sites had thick layers of humified peat (organic matter content 39%–54% and clay+silt content of 46%–61% in the top 20 cm), moderately degraded sites were peaty loams/loamy peats (organic matter content of 21%–34% and clay+silt content of 65%–79% in the top 20 cm), and the most degraded sites were primarily humose‐mineral soils/clays (organic matter content of 11%–18% and clay+silt content of 82%–88% in the top 20 cm). Characterizing each profile allowed us to assess both the underlying edaphic factors as well as the depth of the layers containing peat, rather than relying on the characterization of topsoil alone. Within each farm, two sampling locations with differing levels of soil organic matter content, and thus assumed to represent different degrees of peat loss (the relative degree differed across farms), were identified, and 40‐m transects were set up at each sampling location. Along each transect, we established triplicate sampling points to account for spatial variability. We characterized the soil profiles at each sampling location to confirm they reflected different degrees of peat loss.

### 2.3 Site Ordering

Sites were ordered based on the maximum SOC concentration. Concentration is more reflective of the organic content in a soil whereas stock incorporates bulk density, which can rise with mineral accumulation and thus peat degradation (Figure S2).

### 2.4 Soil Sampling, Peat Depth, Bulk Density and Texture Analysis

Soil samples were collected in June–August of 2023 and 2024. Soils were sampled to 1 m in 2023 to assess peat depth and determine soil types (Figure S1), and to 40 cm in 2024 to conduct biogeochemical measurements, analyzed in two 20‐cm increments (basis of the results section). All soils were sieved to < 2 mm and stored at 4°C until further processing. Subsamples were either frozen at −20°C, freeze‐dried and frozen at −20°C, or air‐dried for ambient storage. To determine the dry bulk density of the topsoil, intact soil cores of a known volume were extracted from the 0–20 cm soil layer in triplicate at each site in July 2024. We built a statistical model to estimate bulk density in the 20–40 cm layer using the 0–20 cm data. Specifically, we fit a partial least squares regression using the sand, silt, and clay content, mineral fraction of soil, and total SOC vs. bulk density relationships in the 0–20 cm data. The first three components explained 85% of the observed variance. This was then applied to the 20–40 cm layer using the measured predictor variables (texture, mineral fraction, and total SOC).

To characterize the sites, we conducted particle‐size analysis following the micro‐pipette method (Miller and Miller 1987) to determine the percentage clay, silt, and sand of dried soil samples. We also measured soil organic matter content using the standard loss‐on‐ignition method with oven dried (60°C) samples combusted at 550°C for 3 h within a furnace (Nelson and Sommers 1982), the recommended conditions for these samples (Hoogsteen et al. 2015). Dried soil samples had been pretreated for carbonate removal prior to analysis using a “no‐leach” method. Specifically, samples were decarbonated by treatment with 0.2 M HCl in sealed exetainers. Following decarbonation, additional 0.2 M HCl was added, and headspace gases were analyzed using a non‐dispersive infrared (NDIR) CO_2_ sensor to confirm complete carbonate removal.

### 2.5 Soil Fractionation, Mineral‐Associated Organic Carbon and Radiocarbon ( ^14^C)

A three‐gram subsample of sieved (< 2 mm) and air‐dried soil collected during the Summer 2024 sampling campaign was used for density fractionation using 1.85 g/cm^3^ sodium polytungstate (SPT) to separate the soil into the heavy and light fractions (i.e., MAOM and POM, respectively) (similar to Leuthold et al. 2024 but optimized for our soils). We verified the SPT solution's density using a hydrometer. We dried soils in the oven at 35°C until at a constant weight. We ashed 47 mm Whatman GF/F filters at 550°C for 4 h, and then weighed them after they cooled.

We added the 3 g subset to 15 mL of SPT. We did not add glass beads because soils were pre‐sieved and did not have noticeable aggregates. The mixers were shaken on a reciprocal shaker at 135 rotations per minute for 3 h; after 1.5 h, the tubes were rotated. The suspensions were then centrifuged for 30 min (at 2330 relative centrifugal force). The supernatant containing the light fraction (density less than 1.85 g/cm^3^) was collected by vacuum filtration with pipetting to avoid losses along the side of the filter. The light fraction was rinsed thoroughly with deionized water (400–500 mL), dried at 50°C to constant weight and then ground with a mortar and pestle. The remaining heavy fraction was washed to remove residual SPT by resuspending the pellet in deionized water by shaking vigorously, centrifuging for 25 min (at 2330 relative centrifugal force), and decanting the supernatant; this rinse was repeated twice more. Soil fractions were dried at 50°C then ground using a ceramic pestle and mortar. Our proportion recovered, calculated on all samples, was 94%.

We did not measure DOC losses during the fractionation process. However, the relative concentration of DOC illustrated concentrations equivalent to < 1% of total SOC in the sample being extracted (*data not presented*). Thus, we assume it is a minimal loss pathway.

All ^14^C fractions were processed using equipment that was decontaminated by either decon 90 (Decon Laboratories Ltd., UK) or by baking in a muffle furnace at 550°C for a minimum of 3 h. A subsample of each fraction was shipped to the NERC Environmental Isotopic Facility (NEIF) in East Kilbride, Scotland, UK, for radiocarbon analysis using previously published methods (Ascough et al. 2024). Samples were decarbonated using the same method described above. The samples were then dried, retaining all material including mobile organic components. Total carbon from a known weight of the pre‐treated sample was recovered as CO_2_ by heating with CuO in sealed quartz tubes. The resulting CO_2_ was converted to graphite by Fe/Zn reduction for radiocarbon measurement.

Samples were also weighed and sealed in tin capsules for measurement of % C and % nitrogen using a Costech Elemental Analyser coupled with a Thermo DELTA V mass spectrometer via a Conflo IV in continuous flow mode at the University of Cambridge.

### 2.6 In Situ Soil Incubations and Determination of Carbon Losses

We measured microbial respiration and C mineralization rates during incubations of soils at optimal moisture conditions (60% WHC) over 126 days. 50 g sieved (< 2 mm) soils were placed in 0.25 L glass jars with screw‐top airtight lids customized with rubber septa. Jars were kept with the lids slightly ajar except during the time when rates were measured. Jars were closed for 2‐h intervals on days 1, 4, 10, 17, 24, 38, 52, 73, and 126. We determined the 2‐h closing interval by conducting several tests varying the interval to ensure the CO2 accumulation was linear. Headspace CO_2_ concentration was < 1000 ppm, and thus we do not expect it to impact microbial activity.

Headspace CO_2_ concentration inside the jar was measured at the start and end of each incubation using a gas‐tight syringe (Hamilton, USA) with an infrared gas analyzer modified for static injection sampling (EGM‐5, PP Systems, USA). When not being measured, the jars were kept in the dark at 20°C. We calculated cumulative C respired (mg CO_2_‐C/g‐soil) on day 126 by averaging the respiration rate between adjacent measurement dates, multiplying the average by the interval between measurements, and then summing. We sampled close together when the change through time was exponential to linearize the change.

### 2.7 Data Analysis

#### 2.7.1 Estimating Mineral Capacity and Deficit

We fit a quantile regression between clay + silt content and mineral‐associated organic carbon to estimate the *maximum* potential mineral sorption capacity in these soils. We fit both a 95th and 99th quantile, with the intercept forced through the origin, consistent with past studies (Georgiou et al. 2025). The mineral C deficit was then calculated by taking the clay + silt content of a sample, predicting the maximum potential mineral sorption based on the above quantile regression, and subtracting the measured MAOC.

#### 2.7.2 Testing for Changes Across the Peat‐Degradation Gradient

Sites spanned peat depths from 0 to 109 cm, with the “wasted peat” sites consisting of those with < 40 cm of peat. We rank‐ordered the sites based on their total SOC for display in the below figures. For statistical tests, we first analyzed whether a response variable differed among sites. We also tested whether there were relationships between peat depth in a site and a response variable. To do this, we fit mixed‐effects models for each response variable as a linear function of peat depth with site as a random intercept to account for the replicate sampling points within a site. Analyses were done in R using the package *lme4* (Bates et al. 2015).

#### 2.7.3 Decomposition Assays

We assessed the potential for carbon losses using a four‐month decomposition assay in the lab (described above). To test whether decomposition rate was related to the composition of SOC, we regressed the fluxes with total POC, MAOC, and proportion of MAOC (MAOC: POC). We conducted this analysis for fluxes at each sampling period, as well as the cumulative flux. Respiration data were log transformed prior to analysis. We also regressed decomposition rate with total carbon stocks. We analyzed respiration rate on a per‐soil mass basis because relativizing it to a per‐SOC value would confound the regressions against POC and MAOC; in other words, MAOC and POC sum to SOC and thus the predictor and predicted variables would not be independent.

#### 2.7.4 Radiocarbon‐Based Turnover Time

To estimate soil organic matter turnover times of the MAOM and POM fractions, we implemented a time dependent steady state radiocarbon modelling framework using Equation (1). Turnover time was defined as the inverse of mean residence time and reflects the average duration that carbon persists within a given pool prior to loss (Trumbore 2000). Radiocarbon values, expressed as absolute fraction modern, together with the proportional carbon contribution of each density fraction, were used to reconstruct bulk soil radiocarbon values via mass balance, since bulk radiocarbon measurements were not available. We assumed DOC losses were minimal. This proportional summation of MAOM and POM fraction modern values provided the bulk constraint required for model calibration.

Turnover time modelling was conducted independently for each individual fraction sample, and the samples within plots were subsequently averaged to obtain site‐level values. Model calibration was performed by iteratively adjusting pool‐specific turnover times until modeled bulk fraction modern values converged with mass balance derived estimates, following established optimization approaches (Gaudinski et al. 2000; McFarlane et al. 2012; Moss et al. 2025; Torn et al. 2002). Atmospheric radiocarbon inputs were assembled from published records (Hua et al. 2022; Hua and Barbetti 2004; Levin and Kromer 2004; Stuiver et al. 1998). A one‐pool model structure was used under the assumptions of steady‐state carbon dynamics and isotopic homogeneity within each pool, with Equation (1) describing soil organic matter turnover time dynamics (Torn et al. 2009).

(1) $$F\prime_{\mathrm{SOM}\;\left(t\right)}=\left[I\times F\prime_{\mathrm{atm}\;\left(t-1\right)}\right.+C_{\left(t-1\right)}F\prime_{\mathrm{SOM}\;\left(t-1\right)}\left.\left(1-k-\lambda \right)\right]/\left[C_{\left(t\right)}\right]$$

where *F*′ = (Δ^14^C × 1000^−1^) − 1 (or absolute fraction modern); *I* = inputs of C to a given SOM pool or fraction (g C m^−2^ y^−1^) which are calculated by 1/turnover time; *C* = Stock of C for the given SOM pool (g C m^−2^); *k* = Decomposition rate constant of the given SOM pool (year^−1^); *F′*_atm_ = the ∆^14^C value of atmospheric CO_2_; *F′*_SOM_ = the ∆^14^C value of the given carbon pool; *λ* = radioactive decay rate of ∆^14^C (year^−1^); and *t* = year in which calculation is being performed.

## 3 Results

### 3.1 Organic Carbon Loading on Minerals Displays a Parabolic Relationship With Degradation

**FIGURE 1:**
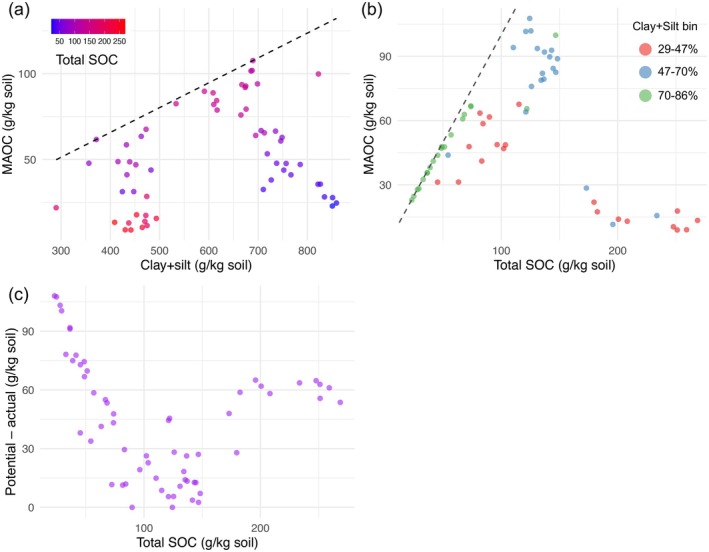
Mineral content and soil organic carbon create a parabolic relationship with mineral‐associated organic carbon (MAOC). (a) maximum‐observed capacity for sorption to minerals determined via a scatter plot between the clay + silt content and MAOC concentration with points colored by total soil organic carbon (SOC) content (g‐C/kg‐soil). Maximum potential organic carbon loading on minerals predicted via quantile regression using a 99th percentile (dashed line; slope corresponds to a carbon loading of 166 mgC/g clay+silt minerals), with a 95th percentile for comparison (152 mgC/g clay+silt minerals) using methods from (Georgiou et al. 2025). (b) scatter plot between total SOC and MAOC, which represents the proportion of SOC that is mineral‐associated. Dashed line is the 1:1 line. (c) deficit for MAOC accrual—calculated as the difference between the actual MAOC concentrations and the maximum potential (as estimated by the quantile regression in panel a) plotted as a function of total SOC. Points include all sample depths.

The amount of carbon associated with minerals peaked at intermediate SOC and mineral content (Figure 1). Specifically, there was a parabolic relationship between the concentration of MAOC and the concentration of minerals (Figure 1a), such that the MAOC was lowest in soils with both very high and very low clay and silt contents (Figure 1a,b). This was partly explained by the bulk SOC concentration in the soil sample: the highest SOC samples had the lowest concentrations of MAOC.

As the mineral content (clay + silt) increased from 46% to 79%, so did the proportion of SOC that was MAOC from 4% to 48%. But in the samples with very high mineral content (> 88%), the concentration of MAOC declined with increasing mineral content despite nearly all SOC being in the MAOC fraction.

The MAOC deficit—determined by subtracting actual MAOC concentrations from the maximum potential calculated via quantile regression (Figure 1a)—was lowest at intermediate SOC values, but increased with decreasing SOC (increasing degradation of peat; Figure 1c). At the high end of the SOC gradient (least degraded peat), there is also a high deficit—these soils still have mineral contents of ca. 30%, but most SOC is POC. We evaluate the processes behind why these high SOC soils do not have high MAOC below, specifically whether decomposition has not sufficiently led to the formation of MAOC.

### 3.2 Peat Degradation Leads to SOC Losses but Primarily via POC

Evaluating the total SOC stocks across the peat‐degradation gradient revealed a large loss of total SOC as peat degraded from the least disturbed site with 266 tC/ha to a “wasted peat” with 71 tC/ha in the top 20 cm (Figure S3; differences among sites: *F*_9,20_ = 69, *p* < 0.001). Losses in the 20–40 cm layer were similarly large, decreasing from 265 tC/ha to 54 tC/ha (Figure S3; differences among sites: *F*_9,20_ = 47, *p* < 0.001). The total SOC stocks were strongly correlated with variation in peat depths in the whole profile as a proxy for degradation among sites (mixed‐effects model with site as a random intercept and peat depth as a fixed effect, 0–20 cm: *t* = 4.038, *p* = 0.004; 20–40 cm: *t* = 4.342, *p* = 0.002; Figure S4). Consequently, there was a clear gradient in SOC losses with peat degradation across the sites.

**FIGURE 2:**
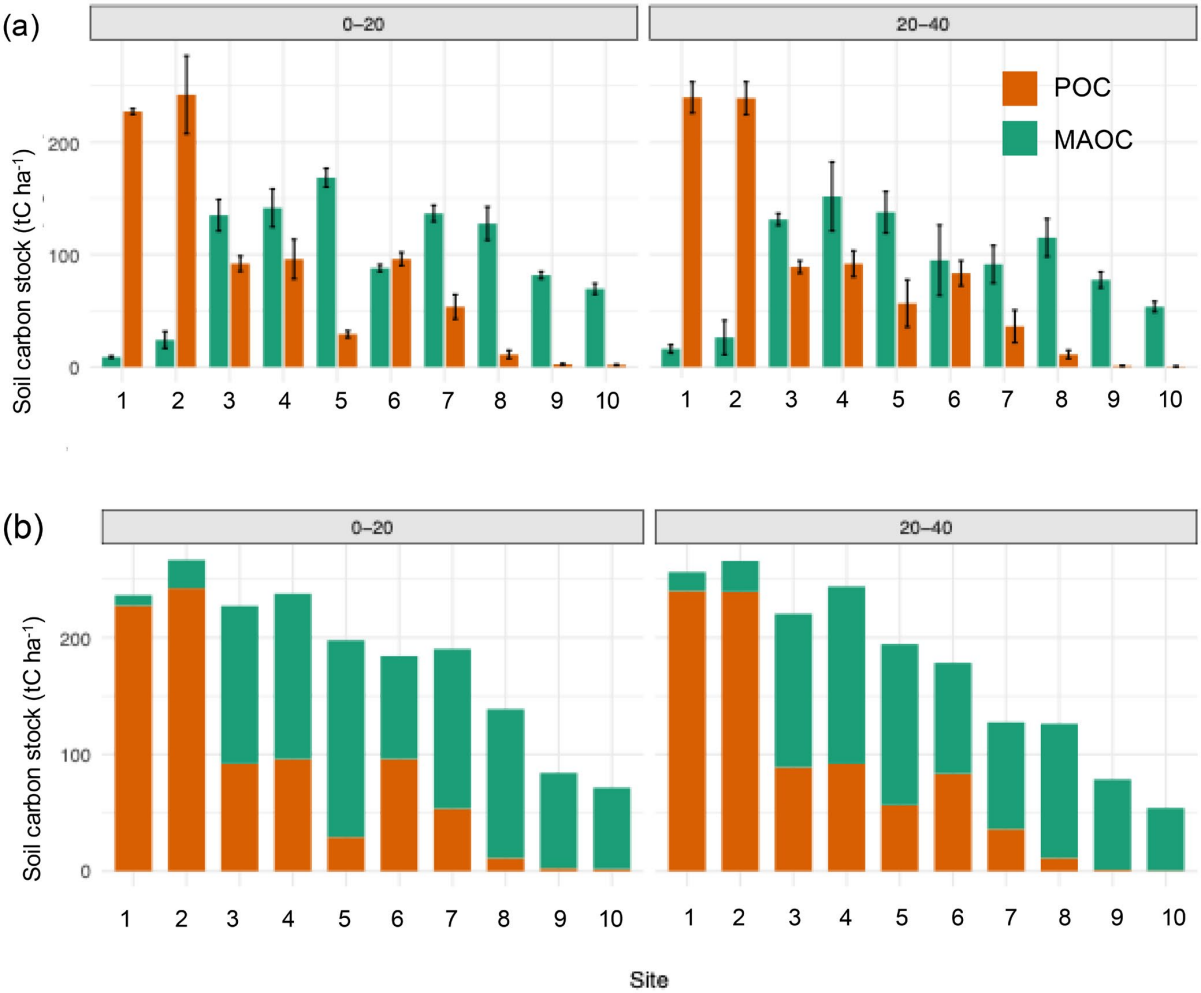
Losses of particulate organic carbon are partly offset by gains in mineral‐associated organic carbon. Stocks of soil organic carbon partitioned between particulate organic carbon (POC) and mineral‐associated organic carbon (MAOC) fractions and by soil depth. (a) total stocks for each fraction separately with bars representing the means and error bars are standard deviations, *n* = 3 within each site. (b) total stocks with the fractions stacked based on the averages across plots within sites. Sites are ordered by descending levels of soil organic carbon concentrations, with the above being the stocks. Facets are the soil depths in centimeters. The numbers next to the sites refer to the two locations within each site.

However, the two soil fractions had contrasting responses. POC declined monotonically from 242 tC/ha to 1.9 tC/ha across the gradient in the top 20 cm (Figure 2; site‐differences: *F*_9,20_ = 131, *p* < 0.001; linear with peat depth: *t* = 3.7, *p* = 0.006). By contrast, MAOC was parabolic, first increasing from 8.9 to 168 tC/ha in the least disturbed to the “wasted peat” sites, then remaining level before finally declining to 70 tC/ha in the lowest SOC site, which we assume is the most degraded (Figure 2; site‐differences: F_9,20_ = 91, *p* < 0.001). Thus, while MAOC never achieved the same overall magnitude as the maximum POC (maximum POC of 242 tC/ha vs. MAOC of 168 tC/ha; Figure 2), it served as a key reservoir during agricultural intensification where nearly all original peat particulates were lost: the site with the lowest SOC had 98% of its SOC as MAOC (69.5 vs. 1.9 tC/ha in MAOC vs. POC, respectively).

We found consistent trends when we combined depths to calculate SOC in the top 40 cm. Soils in the most degraded sites had 406 tC/ha (−77%) less than the least degraded sites that retained a visible surface peat layer. POC accounted for 91% of total SOC in the least degraded but only 2% in the lowest SOC site. Despite the large losses of POC (−479 tC/ha), the gain in MAOC from 25 tC/ha to 123 tC/ha across the same gradient partially offsets the loss of POC.

### 3.3 Formation of MAOC Offsets Losses of POC

If there was no increasing association with minerals, SOC would decline by 240 tC/ha in the top 20 cm across the gradient (using the POC pool changes only), rather than the actual loss of 195 tC/ha. SOC in the 20–40 cm layer exhibited similar trends, where 239 tC/ha of POC was lost but total SOC losses were only 211 tC/ha (Figure 2). Thus, the formation of MAOC reduced losses by ca. 20%. As a result of the MAOC accrual, MAOC comprised 98% of the total SOC pool in the soils of the site at the lowest end of the SOC gradient.

The parabolic relationship of MAOC across the gradient resulted in POC losses being offset even further at intermediate levels of degradation. At sites with intermediate levels of peat degradation, MAOC reached levels of 306 tC/ha when we considered both depths (0–40 cm).

### 3.4 Soils With More POC Experienced Larger Losses From Heterotrophic Respiration

**FIGURE 3:**
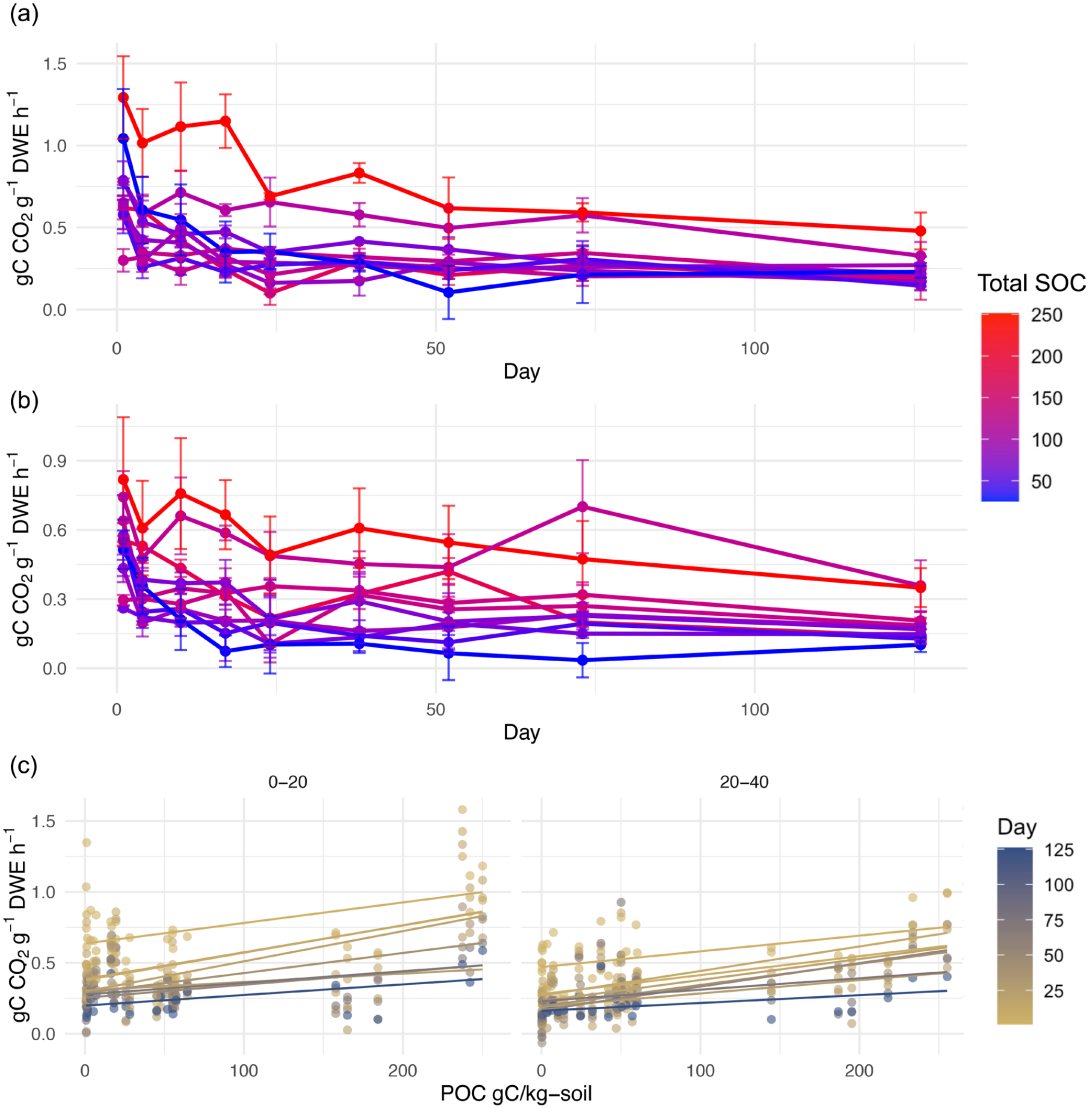
Heterotrophic respiration from lab incubations. CO_2_ production during a four‐month lab incubation re‐scaled to grams of dry soil weight in the 0–20 cm (a) and 20–40 cm (b) layers. Each line represents one site, with the points and error bars representing means and standard deviations. Lines are colored by the soil organic carbon (SOC) content (gC/kg‐soil). (c) regression between respiration and the particulate organic carbon (POC) concentration of the sample. Lines are linear regressions fit for each day.

Sites differed in the cumulative respiration over the four‐month incubation in both soil depths (0–20 cm: *F*_9,20_ = 5.1, *p* = 0.001; 20–40 cm: *F*_9,20_ = 7.6, *p* < 0.001; Figure 3). Cumulative respiration in the site with the highest SOC was approximately double the rate as in the most degraded site (479 vs. 230 mgCO_2_/g‐dry soil, respectively). Although the deeper layer (20–40 cm) had lower overall respiration rates, the cross‐site trends were starker: cumulative respiration was 3.4‐fold higher in the sites with the highest SOC than in the most degraded site (350 vs. 102 mgCO_2_/g‐dry soil, respectively). In both depths, cumulative respiration positively correlated with total SOC (0–20 cm: *F*_1,8_ = 6, *p* = 0.04, *r* ^*2*^ = 0.36; 20–40 cm: *F*_1,8_ = 5.6, *p* = 0.046, *r* ^*2*^ = 0.34). Thus, decomposition of soil tends to be slower in more degraded sites under controlled lab settings.

We next evaluated whether decomposability of the soils across the gradient tracked trends in SOC and the respective fractions. To do this, we analyzed each sampling period separately because of the potential for POC to be “exhausted” during the incubation experiment. When the CO_2_ produced at each of the nine sampling periods (1, 4, 10, 17, 24, 38, 52, 73, 126 days) was analyzed, we found a significant positive relationship (*p* < 0.05) with POC at six of the nine time periods in the top 20 cm (Figure 3). Of the significant relationships, the *r* ^*2*^ ranged from 0.11 to 0.28. Importantly, the last period—126 days—was significant (*F*_1,28_ = 4.7, *p* = 0.039), illustrating that the relationship persists for at least 4 months under lab conditions. Deeper layers displayed even stronger relationships, with all nine sampling periods exhibiting significant relationships with POC (Figure 3).

In contrast, MAOC did not positively correlate with respiration. There were only significant relationships between MAOC and respiration for the first two time periods (1–4 days), and these had negative slopes (*p* < 0.05) in the top 0–20 cm. In the 20–40 cm layer, there was little relationship between MAOC and respiration, except for the final period where it was positively related (*r* ^2^ = 0.11, *p* = 0.044). Taken together, soils with a greater proportion of C as POC tend to have faster rates of carbon loss via heterotrophic decomposition in lab incubations.

### 3.5 Increasingly Modern Radiocarbon Signatures of MAOC and POC Across Sites

**FIGURE 4:**
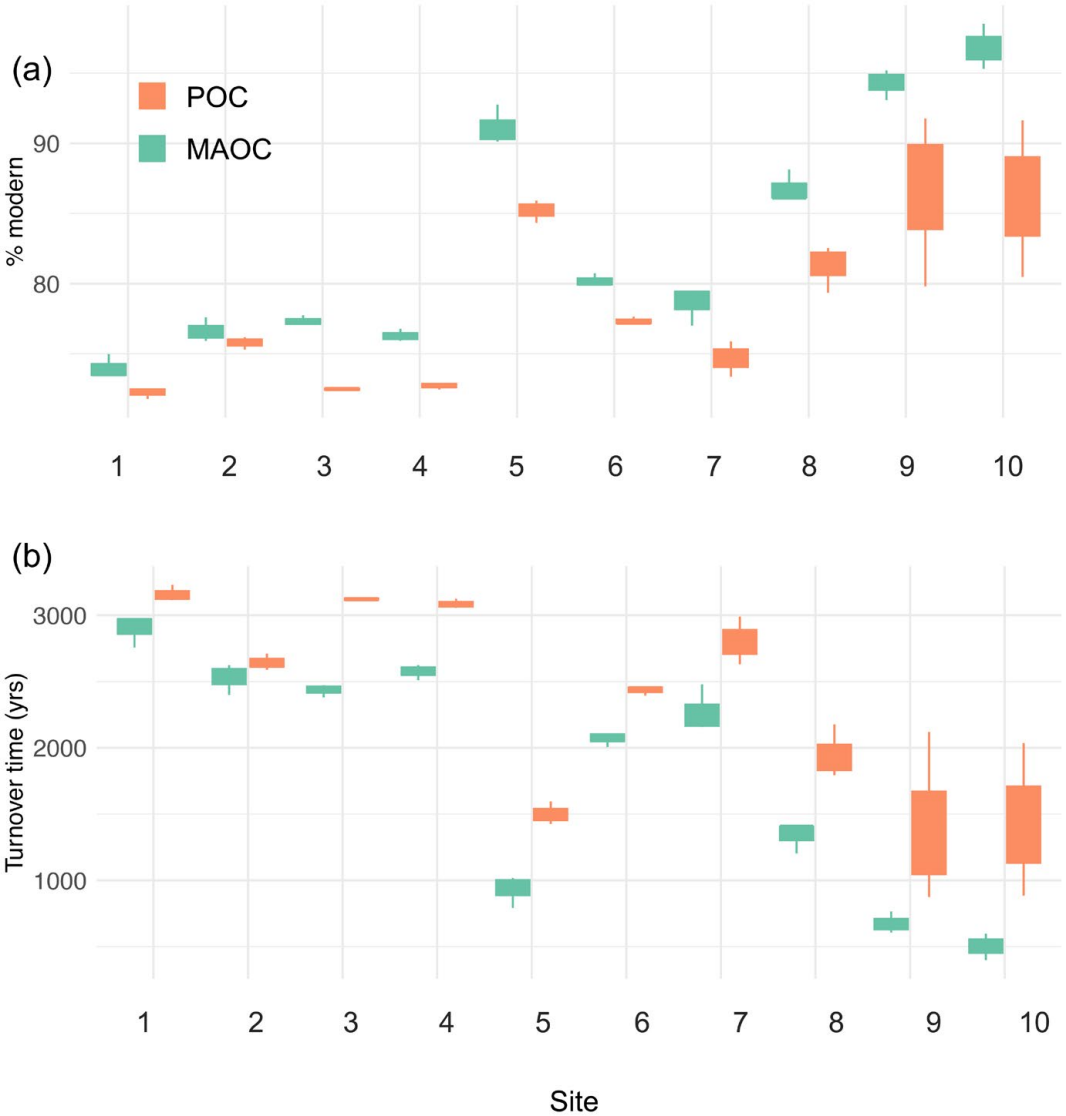
Radiocarbon measurements illustrate that greater degradation results in younger soil organic carbon. Box and whiskers plot of (a) percent of soil C that is modern, split by soil fractions (POC, particulate organic carbon; MAOC, mineral‐associated organic carbon). (b) turnover time of C. Sites are ordered by descending levels of total SOC. Boxes display the quartiles and whiskers are the 10th and 90th percentiles. The numbers next to the sites refer to the two locations within each site. Samples are from the top 0–20 cm.

We found strong differences in the radiocarbon values (percent modern carbon: pMC) of SOC between soil fractions and among sites. Radiocarbon measurements were only made on the 0–20 cm layer. First, the percent modern C was consistently higher in the MAOC versus the POC fraction—namely, 83% ± 8% versus 78% ± 6%, respectively (fraction effect: *F*_1,40_ = 84, *p* < 0.001; Figure 4a; Table S2). The pMC substantially changed across the peat‐degradation gradient (site effect: *F*_9,40_ = 74, *p* < 0.001; Figure 4a), but the change in each fraction was significantly different (site‐fraction interaction: *F*_9,40_ = 2.4, *p* = 0.025). Specifically, the pMC increased with stages of peat degradation, both in the POC and MAOC pools (Figure 4a; bulk SOC in Figure S5). The pMC of POC at the most intact site was 72% ± 0.4%, increasing slightly to 77% ± 0.3% in the intermediate degradation sites, and then increasing substantially in the most degraded sites to 86% ± 6% (Figure 4a). For MAOC, the percent modern increased from 74% ± 0.9% to 80% ± 0.5% to 97% ± 1.6% (Figure 4a). MAOC was always more modern (i.e., pMC closer to 100) than POC, which is consistent with the hypothesis that the microbial pump integrates fresh carbon inputs with processed ancient peat into the MAOC pool.

### 3.6 Turnover Times Were Longer for POC and Decreased With Degradation

Turnover times were longer for POC than for MAOC, averaging 2353 years versus 1822 years across all sites, respectively (fraction effect: *F*_1,40_ = 74, *p* < 0.001). Turnover times of both pools declined across the gradient in peat degradation (site effect: *F*_9,40_ = 59, *p* < 0.001), but did so at different rates (site‐fraction interaction: *F*_9,40_ = 5.5, *p* < 0.001; Figure 4b; Table S2). The turnover time of POC at the most intact site was 3158 ± 62 years, decreasing to 2434 ± 36 years in the intermediate degradation sites, and then decreasing substantially in the most degraded sites to 1434 ± 577 years (Figure 4b). For MAOC, the turnover times decreased from 2898 ± 123 years to 2070 ± 56 years to 502 ± 100 years in the most intact sites, intermediate degradation sites and most degraded sites, respectively (Figure 4b). Across all sites, the turnover time of MAOC remained faster than POC.

Our design assumes that the fields and farms represent an effective “space‐for‐time” substitution along a gradient in the degree to which peat has been lost, which is confirmed from these radiocarbon measurements. Furthermore, the sites that lack any noticeable peat layer (Sites 9–10; Figure S1) and are classified as mineral soils both exhibit similar trends pointing to peat as a source of SOC: the turnover times are relatively long for POC (> 1000 years) and MAOC is always younger than POC (Figure 4, Table S2). Consequently, even though these sites are lacking noticeable peat, they likely had peat at some point.

There was a continuous logarithmic relationship between POC concentrations and stocks with both percent modern C and turnover time of POC. Across all sites, the percent modern C was ca. 85% in plots with the lowest POC stocks (ca. 2 tC/ha) and declined to ca. 75% in plots with the highest POC stocks (ca. 230 tC/ha) (changes were similar for concentrations). The turnover time of POC rose from ca. 1000 years to more than 3000 years in the plots with the lowest to highest POC, respectively. There were no relationships between MAOC concentrations and stocks and both the percent modern and turnover times of MAOC (Figure S6).

### 3.7 Large Potential Accrual From Restoration on Organic Rich Mineral Soils

**FIGURE 5:**
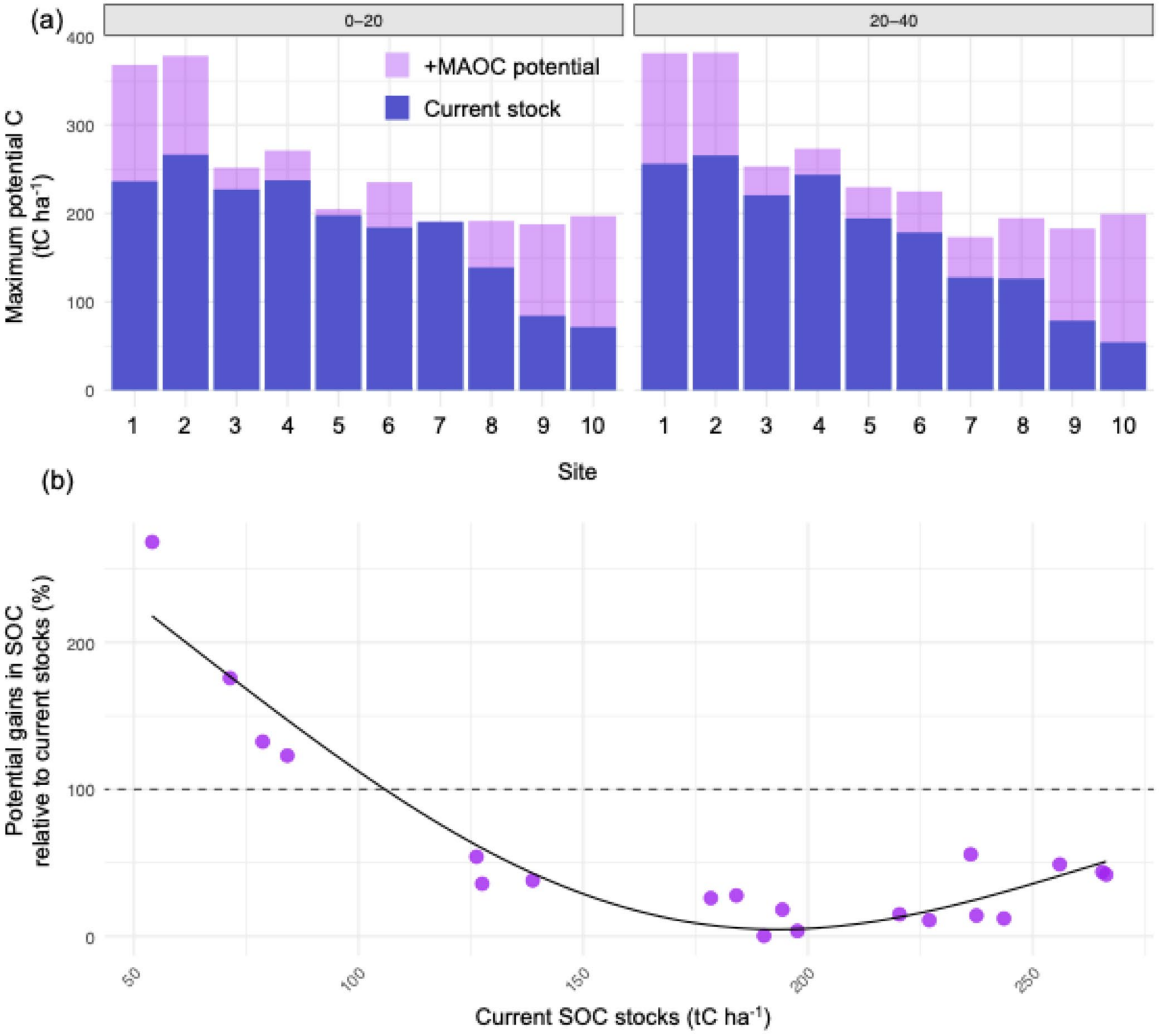
Current soil organic carbon (SOC) stocks and the potential gains if the mineral C deficit was fulfilled. (a) Total SOC stocks (purple) stacked with the potential gains if the mineral‐associated organic carbon (MAOC) potential was achieved. MAOC potential is calculated via the mineral C deficit. (b) relationship between current total SOC and the relative gains in SOC if the MAOC potential was achieved. Curve is fit with a generalized additive model, and the dashed line illustrates 100% (i.e., a doubling).

There was a parabolic relationship between mineral content and potential MAOC sequestration, which revealed two important observations. First, the carbon accrual potential was highest in the least organic rich soils (Figure 5a). These low SOC soils, which had the highest amount of clay and silt content (> 85%), had a high potential maximum accrual of MAOC (+270 tC/ha, 0–40 cm), despite nearly all SOC being MAOC (ca. 98%).

Second, the most organic rich soils also had high potential for MAOC storage (Figure 5b). Even though these were some of the most C rich soils—averaging 20%–28% bulk SOC concentrations—the presence of minerals combined with the lack of historical formation of MAOC (i.e., high mineral C deficit) resulted in high potential. If the MAOC potential was reached, total SOC could increase by an average of +256 tC/ha in the top 0–40 cm. Taken together, high SOC does not preclude these soils from having a high mineral C deficit. Whether this maximum potential accrual is achieved, however, will depend on changes in inputs and losses, which we discuss below.

## 4 Discussion

Our results illustrate that when lowland agricultural peatlands degrade and become intermixed with underlying soil minerals, stabilization of some proportion of the original peat carbon arises via the formation of MAOC. These results shed light on a number of important processes for agriculturally degrading peatlands: (i) this stabilization may help to explain the observed tendency for gaseous CO_2_ emissions to decline on cultivated peatlands as they become increasingly degraded; (ii) it may explain why CO_2_ emissions can continue to be relatively large for many centuries, even when the residual organic layer thickness and SOC content are so depleted that it no longer meets most definitions of organic soil (e.g., D'Acunha et al. 2026), if the MAOC pool is itself unstable under continued intensive cultivation; but also (iii) that there may also be high potential to store more MAOC in the sites towards the end of the agricultural peat‐degradation gradient, despite these sites already having higher SOC than a typical mineral soil, if appropriate management is implemented. The first two points are relevant for understanding GHG emission accounting and their trends over time. The third is relevant for climate change mitigation and nature‐based climate solutions, such as regenerative farming practices developed for mineral soils, but which are increasingly being discussed for wasted peatlands. For example, managing the degraded fields to either increase inputs and/or decrease losses could lead to SOC increases in an already C‐rich soil. These increases would be relative to the current state of the soils, which is far below the total stocks found in the least degraded sites. Consequently, we do not propose that the formation of MAOC can completely offset losses of total SOC or the associated CO2 emissions from peat degradation, but it does serve as a reservoir to help slow and/or reduce the net‐losses. The formation of MAOC could also play a role in reversing some proportion of the historic peat carbon loss in heavily degraded peat landscapes.

Three observations can be made based on the ^14^C results. First, the longer turnover time and low pMC of POC confirm that this is mostly reflective of contributions from the peat‐derived carbon pool. Second, the increase in percent modern C in MAOC illustrates that the accruing SOC is composed of carbon more recently fixed from the atmosphere, consistent with the hypothesis that microbial processing of “fresh” organic matter is what has led to the accumulation of MAOC. Finally, the rising percent of modern C across the gradient illustrates that increasingly more carbon is coming from recent sources, which is expected if peat is lost.

### 4.1 Stabilization of MAOC

The MAOC is likely a key SOC stabilization mechanism, especially in the intermediate stages of peat degradation (once the mineral subsoil becomes intermixed with peat) as MAOC accumulates. First, lab incubations illustrate samples with higher SOC decompose the fastest, but this was due to a positive relationship with POC and not with MAOC (Figure 3). Although lab incubations remove any other important factors for decomposition such as temperature, moisture, and oxygen levels, they are effective at capturing rank‐order differences in SOC losses within sites (Reichstein et al. 2005). The relative decomposability declining as SOC shifts from being POC‐ to MAOC‐dominated during peat degradation is consistent with field‐scale flux measurements. For example, eddy covariance data show that CO_2_ emissions tend to decline along the deep‐shallow‐wasted peat‐degradation gradient (Evans et al. 2016; D'Acunha et al. 2026), which in the past has been attributed to progressive losses of decomposable SOC as degradation proceeds over time under drainage. Our work suggests that this is driven not only by the loss of SOC (via a monotonic decline in peat POC), but also by the transfer of carbon from POC to MAOC.

However, we found that the MAOC pool is not completely stable, with the most degraded sites having high mineral C deficits and low MAOC relative to the intermediate sites that still contain peat but intermixed with minerals. This is consistent with recent work highlighting that components on MAOC can be fast cycling and thus more prone to losses (Jilling et al. 2025). Moreover, turnover times of the MAOC declined by nearly 75% across the sites, becoming increasingly comprised of modern C, which also points to turnover taking place as fresh inputs continue to accumulate and the older peat products degrade. It is important to note, however, that radiocarbon measurements represent an average of the ^14^C content of the pool. Because radiocarbon does not capture the full range of carbon ages present, radiocarbon alone may not fully represent the complexity of MAOC stability.

### 4.2 Peat Degradation, the Microbial Pump and Stabilization of Residual Carbon

Radiocarbon data illustrate that MAOC along the gradient primarily arises from microbial decomposition of peat combined with fresh inputs, resulting in MAOC having more modern C than POC. The long turnover times in POC (> 3000 years in the sites with the most visible peat) are consistent with it having large amounts of C from peat accumulated over the Holocene period (age of upper 20–25 cm layers of nearby peatland deposits are 4400 ± 80 years BP (Hughes 1997)). The finding that MAOC has shorter turnover times than POC across our gradient supports the hypothesis of the microbial pump—whereby microbial decomposition of peat combined with integrating new inputs leads to a greater proportion of modern C than the residual peat (Zhu et al. 2020).

The high mineral C deficits at the most intact sites are likely because the clay/silt layer was hydrologically isolated below the water table hence no flow of dissolved organic matter into that layer and no potential for MAOC accumulation. As the minerals are mixed in and the peat is decomposed, much of the C is respired and lost as CO_2_, but sorption of decomposition products results in the soils approaching their maximum MAOC capacity. The very low mineral C deficits of these intermediate degraded sites speak to the high amount of microbially processed organic matter that is available to form MAOC, despite rapid decomposition and losses of SOC. Consequently, MAOC storage is both a function of mineral content in the soil, as well as the decomposition processes that transfer peat C to mineral surfaces.

Given that MAOC becomes nearly completely modern (> 98%) in the most degraded sites, with turnover times around one‐fifth of values in the most pristine sites, there are likely losses from the MAOC pool as the older SOC is lost. Moreover, the ^14^C measurements suggest that older sources become gradually replaced by newer sources in the POC pool, which then contributes to younger MAOC. Several processes can lead to MAOC being lost, such as simple carbon inputs that stimulate decomposition (Jia et al. 2025; Keiluweit et al. 2015; Li et al. 2021). Given that peat tends to consist of complex organic matter, it is unlikely that peat is priming decomposition and reducing MAOC. Rather, it is likely the agricultural practices and intensive cultivation, such as tilling, that are disrupting the mineral associations (Jilling et al. 2020).

By utilizing the natural gradient in peat mixing with the underlying mineral soils, we provide insight into how MAOC forms and can be subsequently lost. Our results suggest that MAOC is less prone to decomposition, given that it accumulated while POC declined monotonically (Figure 2), was maintained at higher stocks when POC was nearly absent, and that the POC:MAOC ratio was positively correlated with respiration rates. Yet, contrary to theory and past syntheses (Zhou et al. 2024), the turnover time of MAOC was always faster than that of POC (Figure 4). We propose that this is the case because the system is not yet in steady state with the prevailing land use, such that the turnover time is still a legacy of the age of carbon inputs, not a function of the relative decomposability of the different fractions. Despite the POC pool being very small in the most degraded sites, it was still older relative to the MAOC, pointing to a dwindling pool of relic peat‐derived SOC in these soils.

### 4.3 Potential Sequestration via Filling the Mineral C Deficit in Degraded Soils

In degraded soils with low SOC, there is the potential for C sequestration from either the restoration of peatland and formation of new peat if peat‐forming conditions can be reinstated, or the accumulation of MAOC. Given that MAOC formation relies to a large degree on decomposition taking place (and thus putative losses of POC), these two CO_2_ removal strategies are not likely to be completely compatible. In certain geographical settings, however, such as agriculturally important areas or where water resources are limiting, re‐wetting and restoring peat might not be practicable (Zak and McInnes 2022). Furthermore, given that the recovery of the peat carbon sink can take a long time to re‐establish after restoration (Bianchi et al. 2021), managing for MAOC sequestration might be a useful strategy to sequester CO_2_ without bringing cropland out of production. Importantly, however, the accumulated MAOC pool cannot be expected to recuperate the starting SOC pool that would have existed in the pristine peat. This is because both the sheer size of the original carbon pool associated with the several meters of peat that have been lost since drainage, and because much of the MAOC formation depends on the POC source.

The feasibility of realizing the potential MAOC sink can be informed by our parabolic relationship between mineral content and carbon loading. Carbon loading on the minerals—defined as the MAOC per‐mass of silt+clay—ranged from 30 to 160 mgC/g‐clay+silt. However, there was a parabolic relationship, such that intermediate values had the highest C loadings, peaking at ca. 60% clay+silt content, and intermediate total SOC content (Figure 1). Consequently, bringing the most degraded soils back to this intermediate capacity could be one goal. Although we illustrate the potential to mitigate a fraction of historic net‐SOC losses by filling the mineral C deficit (Figure 5), our assessment of maximum observed capacity is on the higher end of previous estimates based on global datasets (Georgiou et al. 2025). We found that the 95th–99th percentiles were 152–166 mgC/g‐clay+silt, compared to 87–120 mgC/g‐clay+silt in a global analysis of mineral soils (Figure 2 and Georgiou et al. 2025). A better understanding of the processes influencing the maximum capacity, such as cation bridging and iron and aluminum oxides, would help to understand why our estimates are on the upper end.

### 4.4 Limitations and Uncertainties

We assume that our gradient is a space‐for‐time representation of how lowland agricultural peat soils change during continuous, long‐term drainage and cultivation. One limitation of this assumption is that the starting peat depth and condition varied as a function of historical peat accumulation processes and rates at each location, and thus any given peat depth and condition may represent different stages in the degradation process (Dawson et al. 2010). All sites have been subject to agricultural drainage, albeit for varying lengths of time, so we could not directly capture the initial period of peatland carbon loss. Although our results suggest MAOC accumulation would have been low during this period while mineral subsoils remained below the water table. Importantly, however, we are less concerned with the temporal dynamics of peat degradation, and more about the processes operating during the latter stage of peat degradation such as how peat SOC might become stabilized. The key assumption there is that the rank‐order based on total soil SOC represents a rank‐order of degradation, which we found strong support for. First, our radiocarbon data illustrate that POC was always older than MAOC, and had exceptionally high turnover times relative to POC in non‐peatland sites (Zhou et al. 2024), characteristic of peat. Second, we also analyzed sites in terms of the soil mineral content, by directly quantifying the key process of peat mixing with minerals, which supported our overall conclusions by comparing among sites. Moreover, the substantial heterogeneity in other underlying edaphic characteristics (61, Figure S5, Table S1) did not obscure the strong trends across sites of POC losses being offset by MAOC gains (Figure 2), microbial processing of peat leading to the accumulation of MAOC (Figure 4), and SOC with more POC being more degradable (Figure 3).

We did not consider other processes that are important for organic matter stabilization. For instance, iron oxides are known to be important for MAOC formation in mineral soils (Kleber et al. 2015; Kögel‐Knabner et al. 2008; Sollins et al. 1996), and have been shown to impact the formation and losses of SOC when peat is intermixed with minerals (Anthony and Silver 2020; Qin et al. 2022). In drained soils, iron (Fe) oxides can lead to the formation of MAOC, but under changing redox conditions (e.g., arising from re‐wetting) can stimulate losses (Anthony and Silver 2020). Our soils have a diverse composition of underlying edaphic properties (Figure S1), and thus metal oxides may help explain variability among sites.

## 5 Conclusions

Our findings illustrate that the decomposition of peat in combination with mixing with underlying mineral soils leads to the accrual of a proportion of the original peat carbon, along with new plant‐derived carbon, in a soil pool that is more stable than the more microbially accessible humified peat. The sorption to minerals of organic matter mobilized from the peat by microbial decomposition helps to limit overall CO_2_ emissions resulting from peat drainage, although this C sink may be small compared to the overall loss of carbon that follows peat drainage. Furthermore, this buffer can itself be diminished by continued cultivation, ultimately reaching steady state with the inputs and outputs determined by land management practices. Quantifying the mineral C deficit of the soils revealed that stocks in the MAOC pool could be increased by around a further 50% in highly‐degraded peatlands, despite SOC already being substantially high. This presents the opportunity for climate mitigation initiatives for the most degraded agricultural peatlands that increase inputs to soils, which does not necessarily require land to be taken out of production when re‐wetting is not practicable. Importantly, this would only offset a fraction of the carbon released by historic peatland carbon loss, but would nevertheless represent effective CO_2_ removal relative to the highly carbon‐depleted soils in the Fens at present.

## Author Contributions

**Anna Basford:** writing – review and editing, methodology, investigation. **Adam F. A. Pellegrini:** conceptualization, investigation, funding acquisition, writing – original draft, methodology, visualization. **Katerina Georgiou:** writing – review and editing, methodology. **Ross Morrison:** writing – review and editing. **Emma Keerberg:** methodology, investigation. **Romy Copley:** methodology, writing – review and editing, investigation. **David A. Coomes:** writing – review and editing, funding acquisition. **Philippa Ascough:** formal analysis, methodology, data curation, writing – review and editing. **Katy J. Faulkner:** conceptualization, methodology, data curation, investigation, formal analysis, resources, writing – review and editing, project administration. **Kimber Moreland:** formal analysis, writing – review and editing. **Alanie Lapina:** methodology, investigation, writing – review and editing. **Christopher Evans:** writing – review and editing. **Rodney G. O. Burton:** methodology, writing – review and editing.

## Funding

This work was supported by UK Space Agency, UKSAG23_0093‐008; UK Research and Innovation, NE/W00495X/1, BB/Z51634X/1, EP/X042863/1; NERC National Environmental Isotope Facility, 2690.1023.

## Conflicts of Interest

The authors declare no conflicts of interest.

## Supporting information


**Figure S1:** Soil profiles classifying the carbon content, texture and pH for each layer. Profiles are ordered based on the groups of farms they arise from, but the site number corresponding to the figures throughout the manuscript text are included as boxes.
**Figure S2:** Distribution of sites and their organic matter properties based on averaging soil carbon in both 0–20 and 20–40 cm samples. Soil organic carbon concentrations used to rank the sites in order of stage of peat degradation.
**Figure S3:** Total soil organic carbon (SOC) stocks across sites in the two depth layers. Bars are the means and error bars are the standard deviations.
**Figure S4:** Changes in topsoil carbon properties as a function of mean peat thickness in a site based on the soil profiles in Figure S1. The peat thickness is the sum of all peat layers across the total profile (not just the 40 cm sampled). SOC, soil organic carbon; MAOC, mineral‐associated organic carbon. Potential‐actual MAOC is determined from the curves in Figure 1.
**Figure S5:** Percent modern in the bulk SOC based on the weighted proportions of POM and MAOM in the sample and their respective % modern values. Bars are mean and standard deviations.
**Figure S6:** Relationship between the carbon content of the samples (stocks: A, B; concentrations: C, D) and percent modern carbon (A,C) and turnover time (B,D). Data are plotted separately for the particulate organic carbon (POC) and mineral‐associated organic carbon (MAOC). Model fits are linear regressions with a logarithmic model fit and were only significant for the POC fraction.
**Table S1:** Site characteristics in the two depth layers. Names are the locations within the Fens and the numbers within them indicate the replicate fields. % sand, silt and clay are per‐mass. Bulk SOC is in percentage, peat depth is in meters and is of the entire profile (hence the same value for both depths), LOI is loss on ignition for total organic matter content in %.
**Table S2:** Radiocarbon‐based measurements of turnover time and percent modern in the different soil fractions across sites. Names are the locations within the Fens and the numbers within them indicate the replicate fields.

## Data Availability

All data used in the manuscript can be freely accessed via Figshare DOI: http://doi.org/10.6084/m9.figshare.32768922.

## References

[gcb70985-bib-0001] Abramoff, R. Z. , K. Georgiou , B. Guenet , et al. 2021. “How Much Carbon Can Be Added to Soil by Sorption?” Biogeochemistry 152, no. 2–3: 127–142. 10.1007/S10533-021-00759-X.

[gcb70985-bib-0002] Angst, G. , K. E. Mueller , K. G. J. Nierop , and M. J. Simpson . 2021. “Plant‐ or Microbial‐Derived? A Review on the Molecular Composition of Stabilized Soil Organic Matter.” Soil Biology and Biochemistry 156: 108189. 10.1016/J.SOILBIO.2021.108189.

[gcb70985-bib-0003] Anthony, T. L. , and W. L. Silver . 2020. “Mineralogical Associations With Soil Carbon in Managed Wetland Soils.” Global Change Biology 26, no. 11: 6555–6567. 10.1111/GCB.15309. 32780521

[gcb70985-bib-0066] Ascough, P. , N. Bompard , M. H. Garnett , et al. 2024. “ ^14^C Measurement of Samples for Environmental Science Applications at the National Environmental Isotope Facility (NEIF) Radiocarbon Laboratory, SUERC, UK.” Radiocarbon 66, no. 5: 1020–1031.

[gcb70985-bib-0004] Bates, D. , M. Mächler , B. Bolker , and S. Walker . 2015. “Fitting Linear Mixed‐Effects Models Using lme4.” Journal of Statistical Software 67: 1–48.

[gcb70985-bib-0005] Bianchi, A. , T. Larmola , H. Kekkonen , S. Saarnio , and K. Lång . 2021. “Review of Greenhouse Gas Emissions From Rewetted Agricultural Soils.” Wetlands 41, no. 8: 108. 10.1007/S13157-021-01507-5.

[gcb70985-bib-0006] Breure, T. S. , D. De Rosa , P. Panagos , M. F. Cotrufo , A. Jones , and E. Lugato . 2025. “Revisiting the Soil Carbon Saturation Concept to Inform a Risk Index in European Agricultural Soils.” Nature Communications 16, no. 1: 1–12. 10.1038/S41467-025-57355. PMC11920252 40102378

[gcb70985-bib-0065] Carlson, K. M. , J. S. Gerber , N. D. Mueller , et al. 2017. “Greenhouse Gas Emissions Intensity of Global Croplands.” Nature Climate Change 7, no. 1: 63–68.

[gcb70985-bib-0007] Chang, Y. , N. W. Sokol , K. J. van Groenigen , et al. 2024. “A Stoichiometric Approach to Estimate Sources of Mineral‐Associated Soil Organic Matter.” Global Change Biology 30, no. 1: e17092. 10.1111/GCB.17092. 38273481

[gcb70985-bib-0008] Conchedda, G. , and F. N. Tubiello . 2020. “Drainage of Organic Soils and GHG Emissions: Validation With Country Data.” Earth System Science Data 12, no. 4: 3113–3137. 10.5194/ESSD-12-3113-2020.

[gcb70985-bib-0009] Cotrufo, M. F. , M. G. Ranalli , M. L. Haddix , J. Six , and E. Lugato . 2019. “Soil Carbon Storage Informed by Particulate and Mineral‐Associated Organic Matter.” Nature Geoscience 12, no. 12: 989–994. 10.1038/s41561-019-0484-6.

[gcb70985-bib-0010] D'Acunha, B. , C. D. Evans , A. Bodo , et al. 2026. “Drained Agricultural Peatlands as Persistent Carbon Sources: Implications for Carbon and Water Use Intensity in Food Production.” Global Change Biology 32, no. 3: e70796. 41801233 10.1111/gcb.70796 PMC12970578

[gcb70985-bib-0011] Dawson, Q. , C. Kechavarzi , P. B. Leeds‐Harrison , and R. G. O. Burton . 2010. “Subsidence and Degradation of Agricultural Peatlands in the Fenlands of Norfolk, UK.” Geoderma 154, no. 3–4: 181–187. 10.1016/J.GEODERMA.2009.09.017.

[gcb70985-bib-0012] Department for Environment, Food & Rural Affairs . 2022. “Agri‐Climate Report.” https://www.gov.uk/government/statistics/agri‐climate‐report‐2022/agri‐climate‐report‐2022.

[gcb70985-bib-0013] Evans, C. , R. Morrison , A. Burden , et al. 2016. “Lowland Peatland Systems in England and Wales – Evaluating Greenhouse Gas Fluxes and Carbon Balances.” http://randd.defra.gov.uk/Default.aspx?Menu=Menu&Module=More&Location=None&Completed=2&ProjectID=17584.

[gcb70985-bib-0014] Evans, C. D. , M. Peacock , A. J. Baird , et al. 2021. “Overriding Water Table Control on Managed Peatland Greenhouse Gas Emissions.” Nature 593, no. 7860: 548–552. 10.1038/s41586-021-03523-1. 33882562

[gcb70985-bib-0015] Gaudinski, J. B. , S. E. Trumbore , E. A. Davidson , and S. Zheng . 2000. “Soil Carbon Cycling in a Temperate Forest: Radiocarbon‐Based Estimates of Residence Times, Sequestration Rates and Partitioning of Fluxes.” Biogeochemistry 51, no. 1: 33–69.

[gcb70985-bib-0016] Georgiou, K. , D. Angers , R. E. Champiny , et al. 2025. “Soil Carbon Saturation: What Do we Really Know?” Global Change Biology 31, no. 5: e70197. 10.1111/GCB.70197. 40345163 PMC12062940

[gcb70985-bib-0017] Georgiou, K. , R. B. Jackson , O. Vindušková , et al. 2022. “Global Stocks and Capacity of Mineral‐Associated Soil Organic Carbon.” Nature Communications 13, no. 1: 1–12. 10.1038/s41467-022-31540-9. PMC9249731 35778395

[gcb70985-bib-0018] Hong, C. , J. A. Burney , J. Pongratz , et al. 2021. “Global and Regional Drivers of Land‐Use Emissions in 1961–2017.” Nature 589, no. 7843: 554–561. 10.1038/s41586-020-03138-y. 33505037

[gcb70985-bib-0064] Hoogsteen, M. J. , E. A. Lantinga , E. J. Bakker , J. C. Groot , and P. A. Tittonell . 2015. “Estimating Soil Organic Carbon Through Loss on Ignition: Effects of Ignition Conditions and Structural Water Loss.” European Journal of Soil Science 66, no. 2: 320–328.

[gcb70985-bib-0019] Hooijer, A. , S. Page , J. Jauhiainen , et al. 2012. “Subsidence and Carbon Loss in Drained Tropical Peatlands.” Biogeosciences 9, no. 3: 1053–1071. 10.5194/BG-9-1053-2012.

[gcb70985-bib-0020] Hua, Q. , and M. Barbetti . 2004. “Review of Tropospheric Bomb ^14^C Data for Carbon Cycle Modeling and Age Calibration Purposes.” Radiocarbon 46, no. 3: 1273–1298. 10.1017/S0033822200033142.

[gcb70985-bib-0021] Hua, Q. , J. C. Turnbull , G. M. Santos , et al. 2022. “Atmospheric Radiocarbon for the Period 1950–2019.” Radiocarbon 64, no. 4: 723–745. 10.1017/RDC.2021.95.

[gcb70985-bib-0022] Hughes, P. 1997. “The Palaeoecology of the Fen/Bog Transition During the Early‐ to Mid‐Holocene in Britain [University of Southampton]. In Revista Médica del Instituto Mexicano del Seguro Social (Vol. 44, Number 5).” https://www.redalyc.org/articulo.oa?id=457745535007.

[gcb70985-bib-0024] Jia, J. , G. Zhai , Y. Jia , and X. Feng . 2025. “Fast Decomposition of Nitrogen‐Rich Mineral‐Associated Organic Matter in Soils.” Global Change Biology 31, no. 8: e70448. 10.1111/GCB.70448;ISSUE:ISSUE:DOI. 40831056

[gcb70985-bib-0025] Jilling, A. , A. S. Grandy , A. B. Daly , et al. 2025. “Evidence for the Existence and Ecological Relevance of Fast‐Cycling Mineral‐Associated Organic Matter.” Communications Earth & Environment 6, no. 1: 690. 10.1038/s43247-025-02681-8.

[gcb70985-bib-0026] Jilling, A. , D. Kane , A. Williams , et al. 2020. “Rapid and Distinct Responses of Particulate and Mineral‐Associated Organic Nitrogen to Conservation Tillage and Cover Crops.” Geoderma 359: 114001. 10.1016/J.GEODERMA.2019.114001.

[gcb70985-bib-0027] Kalisz, B. , P. Urbanowicz , S. Smólczyński , and M. Orzechowski . 2021. “Impact of Siltation on the Stability of Organic Matter in Drained Peatlands.” Ecological Indicators 130: 108149. 10.1016/J.ECOLIND.2021.108149.

[gcb70985-bib-0028] Kasimir‐Klemedtsson, Å. , L. Klemedtsson , K. Berglund , P. Martikainen , J. Silvola , and O. Oenema . 1997. “Greenhouse Gas Emissions From Farmed Organic Soils: A Review.” Soil Use and Management 13, no. 4 SUPPL: 245–250. 10.1111/J.1475-2743.1997.TB00595.X.

[gcb70985-bib-0029] Keiluweit, M. , J. J. Bougoure , P. S. Nico , J. Pett‐Ridge , P. K. Weber , and M. Kleber . 2015. “Mineral Protection of Soil Carbon Counteracted by Root Exudates.” Nature Climate Change 5, no. 6: 588–595. 10.1038/nclimate2580.

[gcb70985-bib-0030] Kleber, M. , K. Eusterhues , M. Keiluweit , C. Mikutta , R. Mikutta , and P. S. Nico . 2015. “Mineral‐Organic Associations: Formation, Properties, and Relevance in Soil Environments.” Advances in Agronomy 130: 1–140. 10.1016/bs.agron.2014.10.005.

[gcb70985-bib-0031] Kögel‐Knabner, I. , G. Guggenberger , M. Kleber , et al. 2008. “Organo‐Mineral Associations in Temperate Soils: Integrating Biology, Mineralogy, and Organic Matter Chemistry.” Journal of Plant Nutrition and Soil Science 171, no. 1: 61–82. 10.1002/jpln.200700048.

[gcb70985-bib-0032] Lavallee, J. M. , J. L. Soong , and M. F. Cotrufo . 2020. “Conceptualizing Soil Organic Matter Into Particulate and Mineral‐Associated Forms to Address Global Change in the 21st Century.” Global Change Biology 26, no. 1: 261–273. 10.1111/gcb.14859. 31587451

[gcb70985-bib-0033] Leuthold, S. , J. M. Lavallee , M. L. Haddix , and M. F. Cotrufo . 2024. “Contrasting Properties of Soil Organic Matter Fractions Isolated by Different Physical Separation Methodologies.” Geoderma 445: 116870.

[gcb70985-bib-0034] Levin, I. , and B. Kromer . 2004. “The Tropospheric 14CO2 Level in Mid‐Latitudes of the Northern Hemisphere (1959–2003).” Radiocarbon 46, no. 3: 1261–1272. 10.1017/S0033822200033130.

[gcb70985-bib-0035] Li, H. , T. Bolscher , M. Winnick , M. M. Tfaily , Z. G. Cardon , and M. Keiluweit . 2021. “Simple Plant and Microbial Exudates Destabilize Mineral‐Associated Organic Matter via Multiple Pathways.” Environmental Science & Technology 55, no. 5: 3389–3398. 10.1021/ACS.EST.0C04592. 33587629

[gcb70985-bib-0036] Lloyd, I. L. , V. Thomas , C. Ofoegbu , et al. 2023. “State of Knowledge on UK Agricultural Peatlands for Food Production and the Net Zero Transition.” Sustainability 15, no. 23: 16347. 10.3390/SU152316347.

[gcb70985-bib-0037] McFarlane, K. J. , M. S. Torn , P. J. Hanson , et al. 2012. “Comparison of Soil Organic Matter Dynamics at Five Temperate Deciduous Forests With Physical Fractionation and Radiocarbon Measurements.” Biogeochemistry 112, no. 1: 457–476. 10.1007/S10533-012-9740-1.

[gcb70985-bib-0067] Miller, W. P. , and D. M. Miller . 1987. “A Micro‐Pipette Method for Soil Mechanical Analysis.” Communications in Soil Science and Plant Analysis 18, no. 1: 1–15.

[gcb70985-bib-0038] Moss, A. , D. Clabo , K. Moreland , and R. B. Abney . 2025. “Prescribed Fire and Pine Straw: Soil Carbon Dynamics in Longleaf Pine ( *Pinus palustris* ) Under Two Common Management Strategies.” Catena 261: 109568. 10.1016/J.CATENA.2025.109568.

[gcb70985-bib-0068] Nelson, D. W. , and L. E. Sommers . 1982. “Total Carbon, Organic Carbon, and Organic Matter.” Methods of Soil Analysis: Part 2 Chemical and Microbiological Properties 9: 539–579.

[gcb70985-bib-0039] Page, S. E. , J. O. Rieley , and C. J. Banks . 2011. “Global and Regional Importance of the Tropical Peatland Carbon Pool.” Global Change Biology 17, no. 2: 798–818. 10.1111/J.1365-2486.2010.02279.X.

[gcb70985-bib-0040] Paul, S. , C. Ammann , Y. Wang , C. Alewell , and J. Leifeld . 2024. “Can Mineral Soil Coverage Be a Suitable Option to Mitigate Greenhouse Gas Emissions From Agriculturally Managed Peatlands?” Agriculture, Ecosystems & Environment 375, no. 24: 109197. 10.1016/j.agee.2024.109197.

[gcb70985-bib-0041] Prairie, A. M. , A. E. King , and M. Francesca Cotrufo . 2023. “Restoring Particulate and Mineral‐Associated Organic Carbon Through Regenerative Agriculture.” Proceedings of the National Academy of Sciences of the United States of America 120, no. 21: e2217481120. 10.1073/PNAS.2217481120. 37186829 PMC10214150

[gcb70985-bib-0042] Qin, L. , C. Freeman , Y. Zou , et al. 2022. “An Iron‐Reduction‐Mediated Cascade Mechanism Increases the Risk of Carbon Loss From Mineral‐Rich Peatlands.” Applied Soil Ecology 172: 104361. 10.1016/J.APSOIL.2021.104361.

[gcb70985-bib-0043] Reichstein, M. , J. A. Subke , A. C. Angeli , and J. D. Tenhunen . 2005. “Does the Temperature Sensitivity of Decomposition of Soil Organic Matter Depend Upon Water Content, Soil Horizon, or Incubation Time?” Global Change Biology 11, no. 10: 1754–1767. 10.1111/J.1365-2486.2005.001010.

[gcb70985-bib-0044] Rhymes, J. , E. Stockdale , B. Napier , et al. 2023. “The Future of UK Vegetable Production: Technical Report.” https://www.wwf.org.uk/our‐reports/future‐uk‐vegetable‐production‐technical‐report.

[gcb70985-bib-0045] Rocci, K. S. , J. M. Lavallee , C. E. Stewart , and M. F. Cotrufo . 2021. “Soil Organic Carbon Response to Global Environmental Change Depends on Its Distribution Between Mineral‐Associated and Particulate Organic Matter: A Meta‐Analysis.” Science of the Total Environment 793: 148569. 10.1016/J.SCITOTENV.2021.148569. 34328984

[gcb70985-bib-0046] Rosenzweig, C. , F. N. Tubiello , D. Sandalow , et al. 2023. “Food System Emissions: A Review of Trends, Drivers, and Policy Approaches, 1990–2018.” Environmental Research Letters 18, no. 7: 074030. 10.1088/1748-9326/ACDDFD.

[gcb70985-bib-0047] Schmidt, M. W. I. , M. S. Torn , S. Abiven , et al. 2011. “Persistence of Soil Organic Matter as an Ecosystem Property.” Nature 478, no. 7367: 49–56. 10.1038/nature10386. 21979045

[gcb70985-bib-0048] Schothorst, C. J. 1977. “Subsidence of Low Moor Peat Soils in the Western Netherlands.” Geoderma 17, no. 4: 265–291. 10.1016/0016-7061(77)90089-1.

[gcb70985-bib-0049] Sokol, N. W. , E. D. Whalen , A. Jilling , C. Kallenbach , J. Pett‐Ridge , and K. Georgiou . 2022. “Global Distribution, Formation and Fate of Mineral‐Associated Soil Organic Matter Under a Changing Climate: A Trait‐Based Perspective.” Functional Ecology 36, no. 6: 1411–1429. 10.1111/1365-2435.14040.

[gcb70985-bib-0050] Sollins, P. , P. Homann , and B. A. Caldwell . 1996. “Stabilization and Destabilization of Soil Organic Matter: Mechanisms and Controls.” Geoderma 74, no. 1–2: 65–105. 10.1016/S0016-7061(96)00036-5.

[gcb70985-bib-0051] Stanley, P. L. , C. Wilson , E. Patterson , M. Machmuller , and M. F. Cotrufo . 2024. “Ruminating on Soil Carbon: Applying Current Understanding to Inform Grazing Management.” Global Change Biology 30, no. 3: e17223. 10.1111/GCB.17223. 38454532

[gcb70985-bib-0052] Stuiver, M. , P. J. Reimer , and T. F. Braziunas . 1998. “High‐Precision Radiocarbon Age Calibration for Terrestrial and Marine Samples.” Radiocarbon 40, no. 3: 1127–1151. 10.1017/S0033822200019172.

[gcb70985-bib-0053] Torn, M. S. , A. G. Lapenis , A. Timofeev , M. L. Fischer , B. V. Babikov , and J. W. Harden . 2002. “Organic Carbon and Carbon Isotopes in Modern and 100‐Year‐Old‐Soil Archives of the Russian Steppe.” Global Change Biology 8, no. 10: 941–953.

[gcb70985-bib-0054] Torn, M. S. , C. W. Swanston , C. Castanha , and S. E. Trumbore . 2009. “Storage and Turnover of Organic Matter in Soil.” In Biophysico‐Chemical Processes Involving Natural Nonliving Organic Matter in Environmental Systems, 219–272. John Wiley & Sons, Inc. 10.1002/9780470494950.ch6.

[gcb70985-bib-0055] Torn, M. S. , S. E. Trumbore , O. A. Chadwick , P. M. Vitousek , and D. M. Hendricks . 1997. “Mineral Control of Soil Organic Carbon Storage and Turnover.” Nature 389, no. 6647: 170–173. 10.1038/38260.

[gcb70985-bib-0056] Trumbore, S. 2000. “Age of Soil Organic Matter and Soil Respiration: Radiocarbon Constraints on Belowground C Dynamics.” Ecological Applications 10, no. 2: 399–411.

[gcb70985-bib-0057] Tubiello, F. N. , M. Salvatore , A. F. Ferrara , et al. 2015. “The Contribution of Agriculture, Forestry and Other Land Use Activities to Global Warming, 1990–2012.” Global Change Biology 21, no. 7: 2655–2660. 10.1111/GCB.12865. 25580828

[gcb70985-bib-0058] Wang, X. , B. Helgason , C. Westbrook , and A. Bedard‐Haughn . 2016. “Effect of Mineral Sediments on Carbon Mineralization, Organic Matter Composition and Microbial Community Dynamics in a Mountain Peatland.” Soil Biology and Biochemistry 103: 16–27. 10.1016/J.SOILBIO.2016.07.025.

[gcb70985-bib-0059] Wang, Y. , S. M. Paul , M. Jocher , et al. 2021. “Soil Carbon Loss From Drained Agricultural Peatland After Coverage With Mineral Soil.” Science of the Total Environment 800: 149498. 10.1016/J.SCITOTENV.2021.149498. 34426363

[gcb70985-bib-0060] Wells, C. E. , and B. D. Wheeler . 1999. “Evidence for Possible Climatic Forcing of Late‐Holocene Vegetation Changes in Norfolk Broadland Floodplain Mires, UK.” Holocene 9, no. 5: 595–608. 10.1191/095968399675019770.

[gcb70985-bib-0061] Zak, D. , and R. J. McInnes . 2022. “A Call for Refining the Peatland Restoration Strategy in Europe.” Journal of Applied Ecology 59, no. 11: 2698–2704. 10.1111/1365-2664.14261.

[gcb70985-bib-0062] Zhou, Z. , C. Ren , C. Wang , et al. 2024. “Global Turnover of Soil Mineral‐Associated and Particulate Organic Carbon.” Nature Communications 15, no. 1: 5329. 10.1038/s41467-024-49743-7. PMC11193739 38909059

[gcb70985-bib-0063] Zhu, X. , R. D. Jackson , E. H. DeLucia , J. M. Tiedje , and C. Liang . 2020. “The Soil Microbial Carbon Pump: From Conceptual Insights to Empirical Assessments.” Global Change Biology 26, no. 11: 6032–6039. 10.1111/GCB.15319. 32844509

