# Supplementary material for: Mineral Stabilization Slows Losses of Peatland Carbon Following Long‐Term Drainage for Agriculture
Source: Glob Chang Biol. 2026 Jul 9;32(7):e70985. doi: 10.1111/gcb.70985 (PMC13347282; doi:10.1111/gcb.70985)
Supplement: Supplementary file 1 — Figure S1: Soil profiles classifying the carbon content, texture and pH for each layer. Profiles are ordered based on the groups of farms they arise from, but the site number corresponding to the figures throughout the manuscript text are included as boxes. Figure S2: Distribution of sites and their organic matter properties based on averaging soil carbon in both 0–20 and 20–40 cm samples. Soil organic carbon concentrations used to rank the sites in order of stage of peat degradation. Figure S3: Total soil organic carbon (SOC) stocks across sites in the two depth layers. Bars are the means and error bars are the standard deviations. Figure S4: Changes in topsoil carbon properties as a function of mean peat thickness in a site based on the soil profiles in Figure S1. The peat thickness is the sum of all peat layers across the total profile (not just the 40 cm sampled). SOC, soil organic carbon; MAOC, mineral‐associated organic carbon. Potential‐actual MAOC is determined from the curves in Figure 1. Figure S5: Percent modern in the bulk SOC based on the weighted proportions of POM and MAOM in the sample and their respective % modern values. Bars are mean and standard deviations. Figure S6: Relationship between the carbon content of the samples (stocks: A, B; concentrations: C, D) and percent modern carbon (A,C) and turnover time (B,D). Data are plotted separately for the particulate organic carbon (POC) and mineral‐associated organic carbon (MAOC). Model fits are linear regressions with a logarithmic model fit and were only significant for the POC fraction. Table S1: Site characteristics in the two depth layers. Names are the locations within the Fens and the numbers within them indicate the replicate fields. % sand, silt and clay are per‐mass. Bulk SOC is in percentage, peat depth is in meters and is of the entire profile (hence the same value for both depths), LOI is loss on ignition for total organic matter content in %. Table S2: Radiocarbon‐based measurements of [file GCB-32-e70985-s001.zip › gcb70985-sup-0002-FigureS1-S6-TableS1-S2@Stabilization of degrading peat manuscript revision SUPPLEMENTAL ACCEPTED.docx]

**Supplemental Information**

**Mineral stabilization slows losses of peatland carbon following long-term drainage for agriculture**

Katy J. Faulkner^1^, Katerina Georgiou^2^, David A. Coomes^1^, Philippa Ascough^3^, Anna Basford^1^, Rodney G. O. Burton^4^ , Romy Copley^1^, Emma Keerberg^1^, Alanie Lapina^5^, Kimber Moreland^6^, Christopher Evans^7^, Ross Morrison^8^, and Adam F. A. Pellegrini^1,6*^

Figure S1:

Figure S1: Soil profiles classifying the carbon content, texture and pH for each layer. Profiles are ordered based on the groups of farms they arise from, but the site number corresponding to the figures throughout the manuscript text are included as boxes.

Figure S2:

Figure S2: Distribution of sites and their organic matter properties based on averaging soil carbon in both 0-20 and 20-40 cm samples. Soil organic carbon concentrations used to rank the sites in order of stage of peat degradation.

Figure S3:

Figure S3: Total soil organic carbon (SOC) stocks across sites in the two depth layers. Bars are the means and error bars are the standard deviations.

Figure S4:

Figure S4: Changes in topsoil carbon properties as a function of mean peat thickness in a site based on the soil profiles in Figure S1. The peat thickness is the sum of all peat layers across the total profile (not just the 40 cm sampled). SOC=soil organic carbon; MAOC=mineral-associated organic carbon. Potential-actual MAOC is determined from the curves in Figure 1.

Figure S5:

Figure S5: Percent modern in the bulk SOC based on the weighted proportions of POM and MAOM in the sample and their respective % modern values. Bars are mean and standard deviations.

Figure S6:

Figure S6: Relationship between the carbon content of the samples (stocks: A-B; concentrations: C-D) and percent modern carbon (A,C) and turnover time (B,D). Data are plotted separately for the particulate organic carbon (POC) and mineral-associated organic carbon (MAOC). Model fits are linear regressions with a logarithmic model fit and were only significant for the POC fraction.

Table S1: Site characteristics in the two depth layers. Names are the locations within the Fens and the numbers within them indicate the replicate fields. % sand, silt and clay are per-mass. Bulk SOC is in percentage, peat depth is in meters and is of the entire profile (hence the same value for both depths), LOI is loss on ignition for total organic matter content in %.

| Site # | Site name | Depth (cm) | % sand | % silt | % clay | pH | Bulk SOC (%) | Peat depth (cm) | LOI (%) |
| --- | --- | --- | --- | --- | --- | --- | --- | --- | --- |
| 1 | Pymoor 1 | 0-20 | 1 | 31 | 14 | 6.35 | 27.8 | 77 | 54 |
| 2 | Barway 1 | 0-20 | 20 | 30 | 12 | 7.31 | 20.3 | 109 | 39 |
| 3 | Manea 1 | 0-20 | 8 | 23 | 35 | 6.73 | 15.4 | 54 | 34 |
| 4 | Pymoor 2 | 0-20 | 2 | 55 | 14 | 7.15 | 13.4 | 50 | 30 |
| 5 | Earith 1 | 0-20 | 2 | 11 | 58 | 6.61 | 12.7 | 74 | 29 |
| 6 | Manea 2 | 0-20 | 33 | 37 | 6 | 7.05 | 13.7 | 40 | 24 |
| 7 | Barway 2 | 0-20 | 37 | 38 | 4 | 7.07 | 9.0 | 40 | 21 |
| 8 | Sawtry 1 | 0-20 | 10 | 31 | 42 | 7.04 | 6.8 | 30 | 18 |
| 9 | Earith 2 | 0-20 | 9 | 15 | 61 | 6.50 | 5.0 | 37 | 15 |
| 10 | Sawtry 2 | 0-20 | 6 | 22 | 60 | 7.56 | 3.1 | 0 | 11 |
| 1 | Pymoor 1 | 20-40 | 1 | 34 | 11 | 6.46 | 27.8 | 77 | 53 |
| 2 | Barway 1 | 20-40 | 12 | 35 | 12 | 7.30 | 22.9 | 109 | 42 |
| 3 | Manea 1 | 20-40 | 8 | 24 | 37 | 6.55 | 14.8 | 54 | 31 |
| 4 | Pymoor 2 | 20-40 | 2 | 55 | 19 | 7.21 | 13.9 | 50 | 25 |
| 5 | Earith 1 | 20-40 | 1 | 14 | 54 | 5.92 | 12.8 | 74 | 31 |
| 6 | Manea 2 | 20-40 | 35 | 39 | 6 | 7.18 | 9.7 | 40 | 20 |
| 7 | Barway 2 | 20-40 | 43 | 36 | 6 | 7.12 | 6.6 | 40 | 15 |
| 8 | Sawtry 1 | 20-40 | 8 | 26 | 49 | 7.12 | 6.5 | 30 | 18 |
| 9 | Earith 2 | 20-40 | 13 | 19 | 54 | 6.68 | 4.1 | 37 | 13 |
| 10 | Sawtry 2 | 20-40 | 5 | 22 | 63 | 7.68 | 2.2 | 0 | 10 |

Table S2: Radiocarbon-based measurements of turnover time and percent modern in the different soil fractions across sites. Names are the locations within the Fens and the numbers within them indicate the replicate fields.

| Site # | Site name |  | Fraction | Turnover time mean (yrs) | Turnover time sd (yrs) | Pct modern mean (%) | Pct modern sd (%) |
| --- | --- | --- | --- | --- | --- | --- | --- |
| 1 | Barway | 1 | POM | 2644 | 62 | 75.8 | 0.45 |
| 2 | Pymoor | 1 | POM | 3159 | 62 | 72.2 | 0.40 |
| 3 | Earith | 1 | POM | 1502 | 86 | 85.2 | 0.81 |
| 4 | Manea | 1 | POM | 3122 | 11 | 72.5 | 0.08 |
| 5 | Pymoor | 2 | POM | 3085 | 36 | 72.7 | 0.24 |
| 6 | Barway | 2 | POM | 2802 | 180 | 74.7 | 1.26 |
| 7 | Manea | 2 | POM | 2434 | 36 | 77.3 | 0.27 |
| 8 | Earith | 2 | POM | 1405 | 643 | 86.5 | 6.12 |
| 9 | Sawtry | 1 | POM | 1947 | 202 | 81.3 | 1.69 |
| 10 | Sawtry | 2 | POM | 1434 | 577 | 86.2 | 5.58 |
| 1 | Barway | 1 | MAOM | 2529 | 117 | 76.6 | 0.87 |
| 2 | Pymoor | 1 | MAOM | 2899 | 123 | 74.0 | 0.86 |
| 3 | Earith | 1 | MAOM | 932 | 122 | 91.1 | 1.43 |
| 4 | Manea | 1 | MAOM | 2435 | 48 | 77.3 | 0.37 |
| 5 | Pymoor | 2 | MAOM | 2574 | 59 | 76.3 | 0.44 |
| 6 | Barway | 2 | MAOM | 2270 | 181 | 78.6 | 1.40 |
| 7 | Manea | 2 | MAOM | 2070 | 56 | 80.2 | 0.46 |
| 8 | Earith | 2 | MAOM | 676 | 82 | 94.3 | 1.08 |
| 9 | Sawtry | 1 | MAOM | 1337 | 115 | 86.8 | 1.15 |
| 10 | Sawtry | 2 | MAOM | 503 | 100 | 96.8 | 1.61 |
